# Enhanced Surface-and-Interface Coupling in Pd-Nanoparticle-coated LaAlO_3_/SrTiO_3_ Heterostructures: Strong Gas- and Photo-Induced Conductance Modulation

**DOI:** 10.1038/srep08531

**Published:** 2015-02-23

**Authors:** Haeri Kim, Ngai Yui Chan, Ji-yan Dai, Dong-Wook Kim

**Affiliations:** 1 Department of Physics, Ewha Womans University, Seoul 120-750, Korea; 2 Clean Energy Research Center, Korea Institute of Science and Technology (KIST), Seoul 136-791, Korea; 3 Department of Applied Physics, The Hong Kong Polytechnic University, Hung Hom, Kowloon, Hong Kong, People's Republic of China

## Abstract

Pd nanoparticle (NP) coated LaAlO_3_/SrTiO_3_ (LAO/STO) heterointerface exhibits more notable conductance (*G*) change while varying the ambient gas (N_2_, H_2_/N_2_, and O_2_) and illuminating with UV light (wavelength: 365 nm) than a sample without the NPs. Simultaneous Kelvin probe force microscopy and transport measurements reveal close relationships between the surface work function (*W*) and *G* of the samples. Quantitative analyses suggest that a surface adsorption/desorption-mediated reaction and redox, resulting in a band-alignment modification and charge-transfer, could explain the gas- and photo-induced conductance modulation at the LAO/STO interface. Such surface-and-interface coupling enhanced by catalytic Pd NPs is a unique feature, quite distinct from conventional semiconductor hetero-junctions, which enables the significant conductance tunability at ultrathin oxide heterointerfaces by external stimuli.

The LaAlO_3_/SrTiO_3_ (LAO/STO) heterointerface, consisting of two wide-bandgap insulators, exhibits unexpected high-mobility two-dimensional electron gas (2DEG) behavior, which has generated an intense surge in research activities by the oxide electronics community^1^,^2^,^3^,^4^,^5^,^6^,^7^,^8^,^9^,^10^,^11^,^12^,^13^,^14^,^15^,^16^,^17^,^18^,^19^,^20^,^21^,^22^,^23^,^24^,^25^,^26^,^27^,^28^,^29^,^30^,^31^,^32^,^33^,^34^. Discovery of additional peculiar physical properties of the LAO/STO system, including a metal–insulator transition^2^,^3^,^4^,^5^, superconductivity^6^, magnetic ordering^7^,^8^, thermoelectricity^9^, strong electron correlation effect^10^, and a huge photoresponse^11^,^12^,^13^,^14^,^15^, has continued. Thus, investigations of these intriguing phenomena have surfaced as the most interesting and challenging topics in relevant fields. First, the electronic reconstruction was suggested as the origin of the unexpected 2D-conduction, which can avoid the polar catastrophe caused by residual net charges at the polar-LAO/nonpolar-STO interfaces^1^,^16^,^17^. Later, sophisticated structural and chemical analyses of the samples prepared at various growth conditions have led us to consider other possibilities, such as creation of oxygen vacancies in the STO substrate^18^,^19^,^20^, off-stoichiometry in the LAO layer^21^, interface doping by cation intermixing^22^, and defect generation during strain relaxation^23^,^24^.

Existence of a built-in electric field in LAO is evidence of the electronic reconstruction at the LAO/STO heterointerface^1^. Atomic force microscopy^3^, scanning tunneling spectroscopy^16^, and photocurrent^11^,^12^ measurement results revealed a signature of the internal field. X-ray photoemission measurements, however, have not shown shifts or broadening of core-level spectra that would reflect the uncompensated internal field^25^,^26^. Such a discrepancy suggests that the aforementioned extrinsic effects - oxygen vacancies and cation disorder - at least partially contribute to the 2D conduction.

The LAO/STO heterointerface can be regarded as a capacitor: the insulating LAO layer is sandwiched by the top surface and the LAO/STO interface (and the underlying STO substrate)^27^,^28^,^29^. A nm-thick LAO film possesses large capacitance, and hence a slight change in the free/bound charges at the LAO surface should significantly modulate the carrier concentration at the LAO/STO interface. Surface charge modification, via biased AFM tip scanning^2^,^3^,^4^,^5^ or polar molecule adsorption^30^,^31^, clearly reveals notable interactions between the surface and interface charges of the LAO/STO 2DEG system. Ferroelectric distortion, if any, could further modify the carrier concentration at the LAO/STO interface^11^,^32^. We should note that the conduction band alignment as well as the net charges at the surface and interface determines the potential drop across the LAO layer^27^,^28^. Adding a metal electrode on the surface of the LAO/STO heterostructure makes this point clear: the Schottky barrier height and band offset will affect the band alignment, subsequently influencing the interfacial carrier concentration^11^,^27^,^28^. All these considerations raise following interesting questions. “How can we directly observe the surface-and-interface coupling of the LAO/STO heterointerface?” “Is it possible to enhance the surface-and-interface coupling?” In this work, we demonstrate that simultaneous measurements of the surface potential and interface conductance of the LAO/STO system provide direct evidence of the strong surface-and-interface coupling. Also, we find that a LAO/STO sample coated with Pd nanoparticles (NPs) exhibits an enhanced relationship between the surface potential and interface conductance, compared with the bare sample. These results elucidate the underlying mechanism for remarkable ambient-dependent photoresponse of the Pd-NP-coated LAO/STO samples^15^. This work provides us with valuable insights regarding the 2DEG conduction at the LAO/STO heterointerface and further improvement of novel functional devices based on atomically controlled oxide heterostructures.

Epitaxial LAO thin films with 5 unit cell thickness were grown on TiO_2_-terminated STO substrates by pulsed laser deposition (PLD)^15^,^31^,^33^. After the depositions, Pd NPs were coated on top of the LAO/STO surface using DC magnetron sputtering at room temperature. The NPs are crystallized and the density does not lead to a complete conduction percolation path, as shown in Fig. 1a. Detailed sample preparation procedures and characterization results can be found in earlier publications^15^,^33^. Hereafter the LAO/STO heterostructures without and with the Pd NPs will be called as LAO/STO and Pd/LAO/STO, respectively. Figure 1b schematically illustrates the measurement setup: transport and Kelvin probe force microscopy (KPFM) measurements were simultaneously performed in a glove box while varying the ambient gas environments^5^, such as H_2_(2%)/N_2_(98%), N_2_, and O_2_, and with and without UV light (wavelength: 365 nm and intensity: ≥10 mW/cm^2^) illumination.

Figure 2 shows conductance (*G*) and surface photovoltage (SPV – difference of work function before and after light illumination) of LAO/STO and Pd/LAO/STO as a function of time in different gases, obtained during gas exchange (from air to N_2_, from N_2_ to H_2_/N_2_, and from N_2_ to O_2_) and illumination of UV light (detailed experimental procedures can be found in Method). In all the gases, *G* of the samples increases under the UV light. When the light is off, *G* decreases very slowly. Such a long relaxation time of the photocurrent, lasting over a day, is called persistent photoconductivity (PPC). PPC is believed to be a signature of trap states, mostly located near the surface and the interface of the samples^13^,^14^. Positive SPV (i.e., larger work function in dark) appears in all the cases. When the change in *G* is larger, larger SPV appears. SPV also shows long relaxation behavior like *G*. Such similar tendencies between *G* and SPV imply the existence of a common physical origin in these two quantities. It is also notable that the Pd NPs significantly enhance the gas sensitivity of the LAO/STO heterostructure, as reported earlier^33^. In particular, the gas exchange from N_2_ to H_2_/N_2_ induces a more abrupt conductance change of Pd/LAO/STO.

First, we examine the ambient dependence. Figure 3a and 3b show the ambient dependence of *G* and work function (*W*) of the LAO/STO and Pd/LAO/STO samples in dark, respectively. All the measured *G*_Pd/LAO/STO_ values are smaller than the *G*_LAO/STO _values. As mentioned above, the LAO/STO sample can be regarded as a capacitor. Thus, the change in the potential drop across the LAO layer, *V*', should alter the sheet electron density at interface, *σ*,
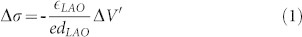
where *ε*_LAO_ is the dielectric constant of LAO, *e* is the unit charge, and *d*_LAO_ is the thickness of LAO^5^,^30^.

The large *W* of Pd (5.6 eV) causes band bending at the Pd/LAO interface^27^,^28^ and the space charge region (SCR) is supposed to be wider than *d*_LAO_ (only ~2 nm), as illustrated in Fig. 4a and 4b. An interfacial layer, which can be formed at two dissimilar metal and oxide layers, is included in Fig. 4a^34^. The Fermi level adjustment at the interface (i.e., band bending) by the Pd contact alters *V*' and consequently varies *W* (Fig. 4a). If the mobility (*μ*) of both LAO/STO and Pd/LAO/STO is assumed to be almost the same at room temperature^18^,^30^, the relation between *G* and *σ* can be obtained,
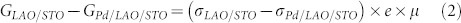
From Eqs. 1 and 2, a following equation can be derived.
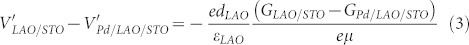
*V*'_LAO/STO_ − *V*'_Pd/LAO/STO_ is estimated to be −0.12 eV in N_2_, from the measured difference in the *G* values.

As shown in the TEM image (Fig. 1a), the diameter of the Pd NPs and the distance between neighboring NPs are less than several nm, and hence KPFM cannot discern the work functions of the NPs and the bare LAO/STO surface. Thus, the measured surface work function of Pd/LAO/STO, *W*_Pd/LAO/STO_, corresponds to the weighted average of *W*_Pd_ and *W*_LAO/STO_: *W*_Pd/LAO/STO_ = *f* × *W*_Pd_ + (1 − *f*) × *W*_LAO/STO_ (*f*: surface coverage of Pd). *f* is estimated to be 0.41: *W*_Pd/LAO/STO_[N_2_] = 4.95 eV, *W*_LAO/STO_[N_2_] = 4.5 eV (both from the data in Fig. 3b), and *W*_Pd_ [N_2_] = 5.6 eV. The estimated *f* seems reasonable, from the TEM image in Fig. 1a and consideration of the band bending around the NPs, as illustrated in Fig. 4b^35^. Therefore the band bending by the Pd NPs explains the results obtained from Pd/LAO/STO and LAO/STO in N_2_: the Pd overlayer increases *V*' and *W*, resulting in decrease of *G*.

We should note that *G*_Pd/LAO/STO_ and *W*_Pd/LAO/STO_ in H_2_/N_2_ are largely different from those in N_2_ and O_2_. Taking hydrogen adsorption into account, two possible processes, i.e., PdH_x_ formation^36^ and dissociate adsorption of H_2_ on the LAO/STO surface^37^ (Fig. 5a), can occur. PdH_x_ has smaller *W* than Pd, lowering *V*' and raising *σ*, according to Eq. (1). Hydrogenation of Pd increases the volume of NP (<2%), which further enhances the conductivity change^36^. (*V*'_Pd/LAO/STO_[H_2_/N_2_] − *V*'_LAO/STO_[N_2_]) is estimated to be −0.07 eV from (*G*_Pd/LAO/STO _[H_2_/N_2_] − *G*_Pd/LAO/STO _[N_2_]), using a relation similar to Eq. (3). The measured *ΔW*_Pd/LAO/STO_ in H_2_/N_2_ and N_2_ (0.75 eV) is much larger than the change of *V*' expected from measured *G*. The electron affinity of LAO will be invariant in any gas, and hence surface dipoles caused by the dissociative H_2_ adsorption should be considered to explain *ΔW*, as illustrated in Fig. 5a. Potential drop by a surface dipole, Δ*ϕ_S_*, is given by a following equation
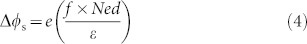
where *f* is the surface coverage, *N* is the number of unit cells per area, *d* is the length of the dipole, *ε* is the dielectric coefficient of the dipole layer^5^,^38^. When *f* of H^+^ is only 4%, Δ*ϕ_S_*[H_2_/N_2_] can be as large as −1 eV. The adsorbed hydrogen atoms donate the carriers to the interface^37^ and increase *G*: the expected amount of *ΔG*/*G* ~ 4% by the hydrogen adsorption is much smaller than the experimental value, 110%. Thus, the band bending alteration by the PdH_x_ formation and the surface dipoles formed by the H adsorption on the LAO/STO surface can mainly contribute to the notable increase of G_Pd/LAO/STO_ and decrease of *W*_Pd/LAO/STO_ in H_2_/N_2_, respectively.

A finite amount of oxygen vacancies should be present in the as-prepared LAO thin film^4^,^12^,^13^,^14^. If oxygen vacancies in LAO can be annihilated through dissociative adsorption of O_2_ (Fig. 5b), *V*' and *W* increase, resulting in decrease of *σ* (Eq. 1)^20^. The experimental data shows that the *G* of LAO/STO and Pd/LAO/STO decreases in O_2_ compared to N_2 _(Fig. 3a). It is noteworthy that the *W* of LAO/STO and Pd/LAO/STO in O_2_ is somewhat larger than that in N_2_. Assuming the oxidation of LAO as the only reason to cause the change in *G*, (*V*'_Pd/LAO/STO_[O_2_] − *V*'_Pd/LAO/STO_[N_2_]) is estimated to be +0.01 eV from (*G*_Pd/LAO/STO _[O_2_] − *G*_Pd/LAO/STO _[N_2_]) using Eq. 3. The measured (*W*_Pd/LAO/STO_[O_2_] − *W*_Pd/LAO/STO_[N_2_]) is +0.11 eV, 10 times larger than the expected difference in *V*'. This suggests that the oxidation of LAO/STO cannot explain the change in *W*. The surface adsorption also can change *W*, as discussed in the H_2_/N_2_ gas data. According to Eq. (4), surface coverage of O_2_^−^ less than 1% can increase *W*_Pd/LAO/STO_ by ~0.1 eV. Oxidation of STO also can decrease *G* in O_2_, which would not vary *W*^39^. Thus, the variation of *G* and *W* in N_2_ and O_2_ can be attributed to oxidation and the surface dipoles caused by dissociated adsorption of O_2_ molecules.

As clearly shown in Fig. 3a and 3b, the gas response of LAO/STO is significantly enhanced by deposition of Pd NPs. The ambient dependence can be explained by several key processes: (1) the reaction of Pd NPs with the surrounding gas molecules and resulting potential profile alteration, (2) surface redox in LAO and change in *V*', and (3) hydrogen and oxygen adsorption at the LAO surface and dipole formation. The catalytic Pd NPs can enhance active dissociation of H_2_ and O_2_ molecules. The dissociated atoms diffuse into the LAO film, promoting adsorption and/or redox on the surface (the so called ‘spillover effect')^40^,^41^. Such a spillover effect contributes to the strong gas-dependent conductance modulation.

The UV-induced conductance change, *ΔG*_light_ = *G*_light_ − *G*_dark_, of the two samples in different gas (obtained from the data in Fig. 2) is compared in Fig. 6a. Figure 6b shows SPV, manifesting the close relationship between *ΔG*_light_ and SPV. This reveals strong surface-and-interface coupling of the LAO/STO heterointerface under UV illumination as well as in dark. *ΔG*_light_ and SPV of Pd/LAO/STO exhibits a more notable ambient dependence, compared with LAO/STO. Research on gas sensor and photodetector applications have shown that the creation of photocarriers, charge transfer from/to adsorbates, work function alteration of metal contacts by reaction, and surface band bending by trapping/detrapping of surface states can influence the bulk and contact resistance of oxide devices^33^,^35^,^40^,^41^. In most of these studies, dominant resistance modification occurs at the surface of oxides directly exposed to the external stimuli and just below the surface. In contrast, the resistance of the LAO/STO heterointerface is determined by the buried interface not the top surface. In this regard, the enhanced gas and UV sensitivity of the Pd/LAO/STO heterointerface is very distinct from those of other usual oxide systems and deserves more attention.

The incident photon energy (3.4 eV) is larger than the bandgap of STO (3.2 eV) but smaller than that of LAO (5.6 eV). Thus, these photons create electron-hole pairs in STO and the electrons move to the potential well at the LAO/STO interface, resulting in Δ*G*_light_ > 0 (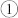 of Fig. 7)^11^,^12^,^13^,^14^. The incident photons also can excite electrons from the Pd NPs, some of which can overcome the potential barrier at the Pd/LAO interface and produce a photocurrent (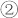 of Fig. 7)^42^. The accumulated carriers at the LAO/STO interface decrease *V*' and *W*_light_, resulting in positive SPV ( = *W*_dark _− *W*_light_), as illustrated in 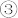 of Fig. 7. As discussed above, Pd NPs can cause upward band-bending in the LAO layer, by which electrons are quickly swept to the interface, increasing Δ*G*_light_ and SPV. We did measure the mobility of our LAO/STO and Pd/LAO/STO samples before and after illumination of UV light by the Hall measurement system using the Van der Pauw configuration at room temperature. The mobility of the samples was almost the same. This suggests that the photo-response in the conductance should be attributed to the change in the carrier density.

In addition, the ambient dependence of Pd/LAO/STO is notable: the smallest *ΔG*_light_ appears in H_2_/N_2_, compared with those in N_2_ and O_2_. As aforementioned, small *W* of PdH_x_ can reduce *V*' in H_2_ containing gas and hence a low internal electric field in LAO would not readily separate photo-generated electron hole pairs, resulting in a weak UV response. The photoresponse in N_2_ and O_2_ also can be compared: *ΔG*_light_ of Pd/LAO/STO in O_2_ is smaller than that in N_2_. SPV of Pd/LAO/STO is almost the same in N_2_ and O_2_. A decrease in oxygen vacancies will decrease *ΔG*_light_ due to a smaller photocurrent contribution from the defect states^12^,^13^,^14^. As discussed above, the O_2_^-^ adsorbates form surface dipoles on the Pd/LAO/STO sample in O_2_ and raise *W*. Some of the photo-excited holes in STO under UV illumination can reach the LAO surface through diffusion, drift, and tunneling^11^, combining with the negatively charged oxygen ions (O_2_^-^) and producing molecular oxygen (O_2_) gas^33^. Such oxygen desorption decreases *W*_light_ and a small *W*_light_ increases SPV. Thus, the difference in N_2_ and O_2_ can be attributed to the photo-induced desorption. These results suggest that the catalytic activity of the Pd NPs plays an important role in ambient-dependent UV photoresponse of LAO/STO, further enhancing the surface-and-interface coupling.

Pd nanoparticle-coated LaAlO_3_/SrTiO_3_ heterointerface (Pd/LAO/STO) exhibits enhanced UV photoresponse compared with a bare sample (LAO/STO). The conductance and work function of Pd/LAO/STO also exhibit a strong ambient dependence in N_2_, H_2_/N_2_, and O_2. _Simultaneous measurements of conductance and surface work function in dark and UV illumination can reveal key physical processes involved in the ambient- and light-dependent response of Pd/LAO/STO samples: PdH_x_ formation, oxygen vacancy annihilation, and surface adsorption/desorption with subsequent charge transfer. In particular, the quantitative analyses manifest the catalytic activity of Pd nanoparticles. These results demonstrate the unique features of the LAO/STO heterointerface, its strong surface and interface coupling and its crucial role in the intriguing transport phenomena.

## Methods

The KPFM measurements were carried out using an atomic force microscopy system (XE-100, Park Systems Co.) with a glove box. Conductive Pt-coated Si cantilevers (NSG10/Pt, resonance frequency: ~240 kHz, NT-MDT) were used for both work function and topography measurements in the noncontact mode. Applied bias to the tip had both ac (frequency: *ω*) and dc components. The lock-in technique allows extraction of the *ω* component of the tip deflection, which is proportional to the electrostatic force between the tip and the sample. Just after each measurement, the work function of the tip was calibrated with the HOPG (highly ordered pyrolytic graphite, SPI Supplies) reference sample. Prior to the measurements, the samples were stored in dark for at least one day in air, whose relative humidity was 20–30%. The detailed experimental procedures for ambient-dependent KPFM measurements are described in our earlier publication (Ref. 5).

For the transport experiments, Al wires were bonded at the sample corner, where the top LAO layer was removed, as well as pads in a home-made PCB by a wire bonder (7476D, West Bond Inc.). The two-probe electrical measurements were performed by a semiconductor parameter analyzer (4156B, Hewlett Packard) simultaneously with the work function measurements in each gas.

The work function and conductance measurements were done in air at first, and then the glove box was purged by N_2_. After the purging for about 10 hours, the measurements were performed while maintaining the flow of N_2 _gas. The identical measurements repeated in H_2_/N_2_ gas in a similar way: ~10 hours of purging and continuing flow of H_2_/N_2_ gas during the measurements. Prior to exchanging gas from H_2_/N_2_ to O_2_, the system was purged by N_2_ for more than 10 hours to remove residual H_2_ gas and then filled with O_2_.

In order to examine photo-induced effects, we used a 10 mW UV light source with wavelength of 365 nm (SUV-S series, UVSMT) equipped with a power control unit. The UV light was delivered by a fiber to the sample and aligned to illuminate the center of the sample by a position-adjustable stand. The measurements in each gas were conducted with and without the UV light. Prior to the measurements, samples were stored in dark for at least one day, in order to exclude the possible influence of persistent photocurrent.

## Author Contributions

H.K. and D.K. performed the measurements and analyzed the data. N.Y.C. and J.D. prepared and characterized the samples. All authors discussed the results and commented on the manuscript.

## Figures and Tables

**Figure 1:**
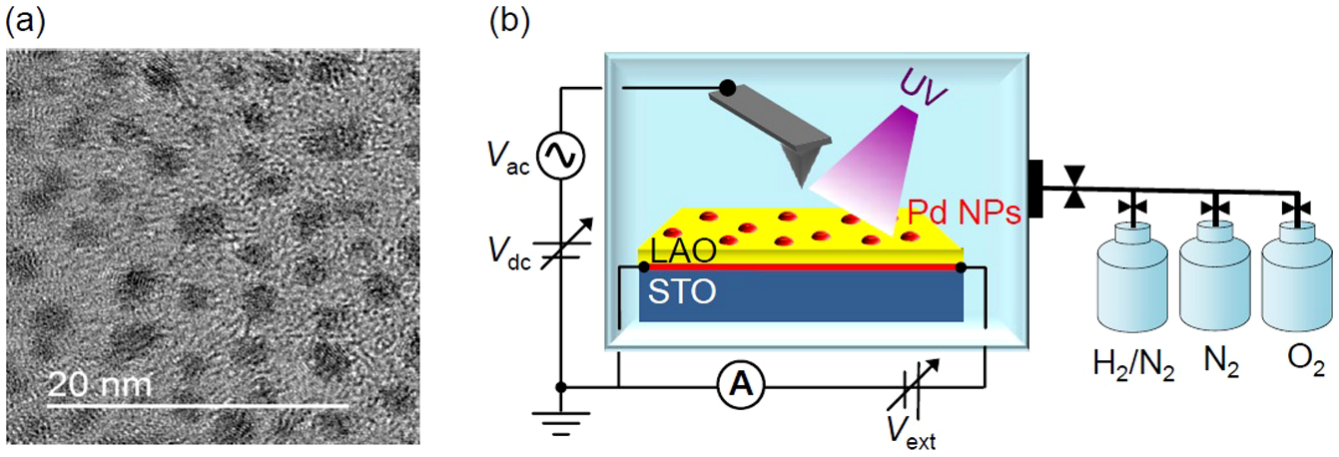
(a) TEM image showing typical size and spatial distribution of Pd NPs prepared on carbon grid and (b) schematic illustration of transport and KPFM measurement set-up.

**Figure 2:**
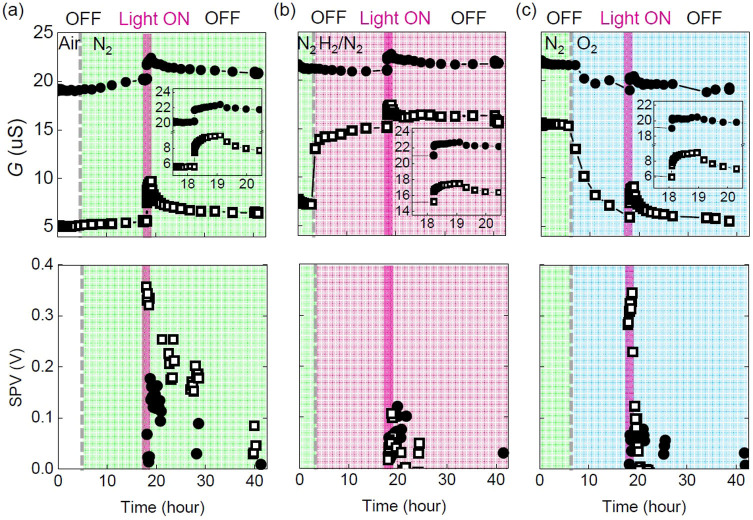
UV response, conductance (*G*) and surface photovoltage (SPV) of LAO/STO (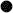) and Pd/LAO/STO (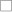) in (a) N_2_, (b) H_2_/N_2_, and (c) O_2_. The gray dashed lines and the purple bars indicate the time when gas exchange starts and the period of UV light illumination (1 hour), respectively. The insets in (a)–(c) clearly show saturation behaviors of *G* under UV illumination.

**Figure 3:**
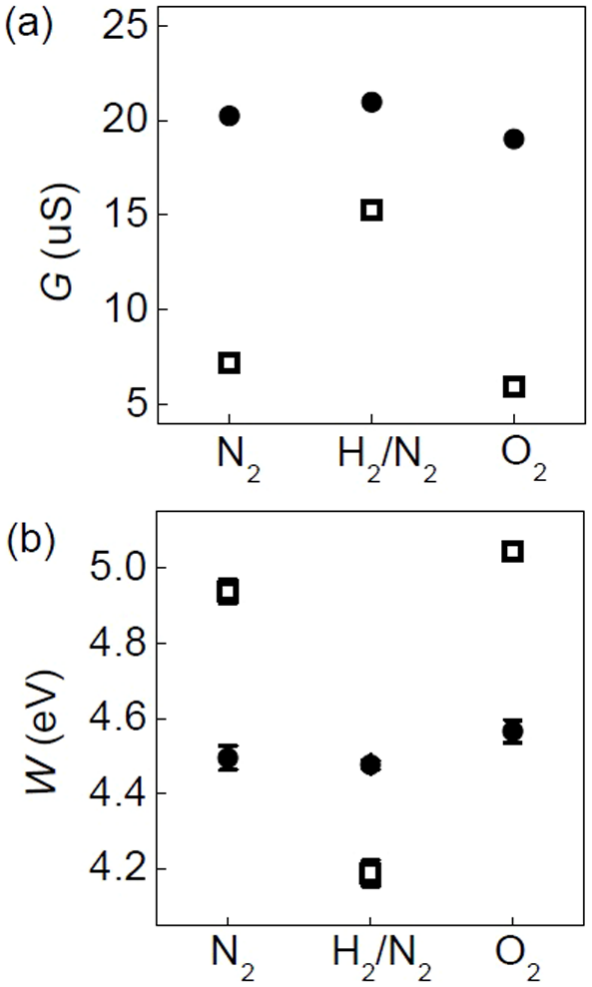
Gas ambient dependence of LAO/STO (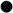) and Pd/LAO/STO (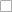) in dark: (a) Conductance, *G*, and (b) surface work function, *W*. Both *G* and *W* in each gas are taken from the data in Fig. 2, just before the light illumination.

**Figure 4:**
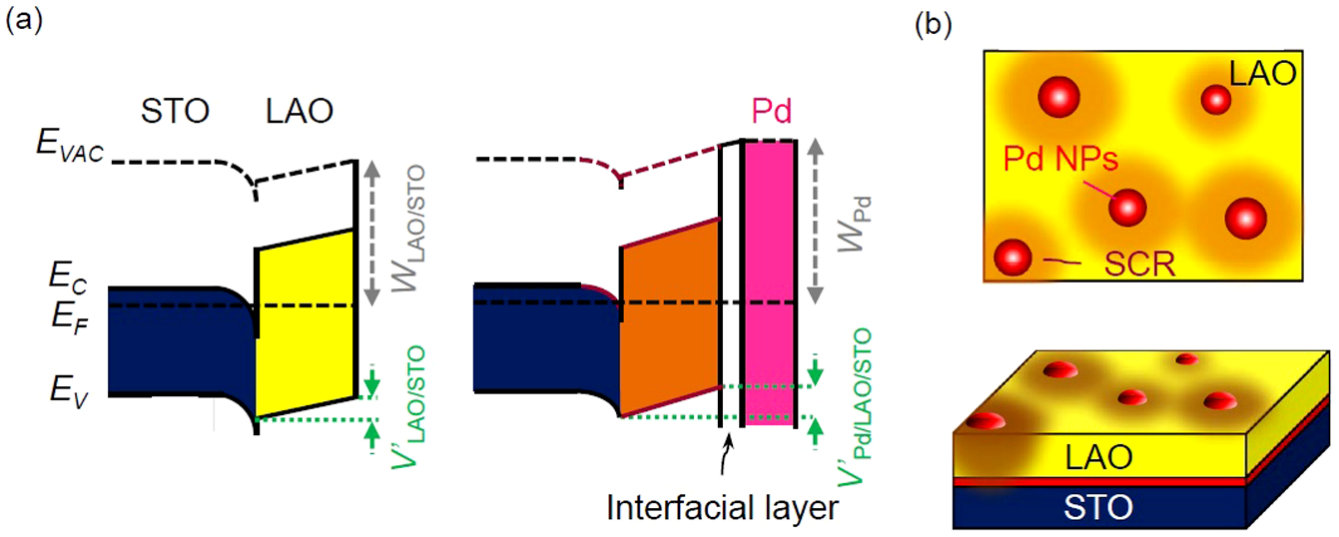
(a) Band diagrams of bare LAO/STO and Pd/LAO/STO. (b) Schematic illustrations of Pd/LAO/STO. Space charge region (SCR) formed around the Pd NP contacts is illustrated in shaded regions.

**Figure 5:**
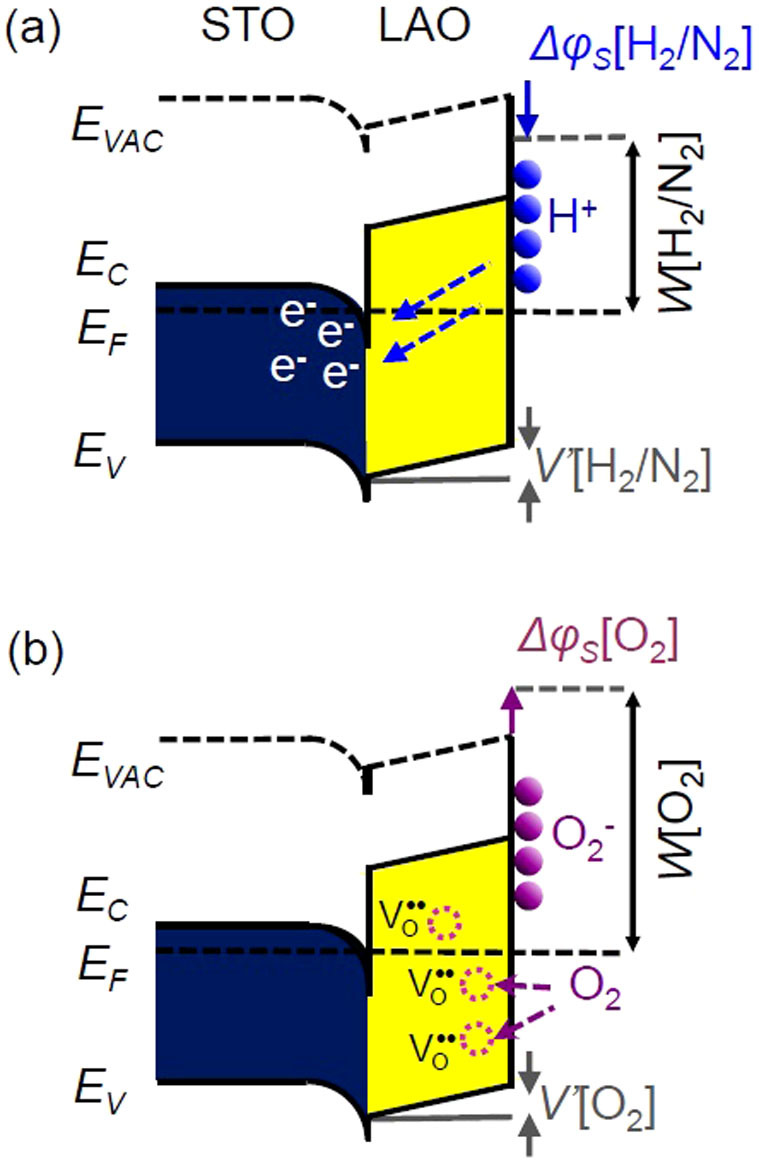
Schematic band diagrams for dissociate adsorption of (a) H_2_ and (b) O_2 _on the LAO/STO surface in dark. e^-^ and 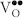 denote electron and oxygen vacancy, respectively.

**Figure 6:**
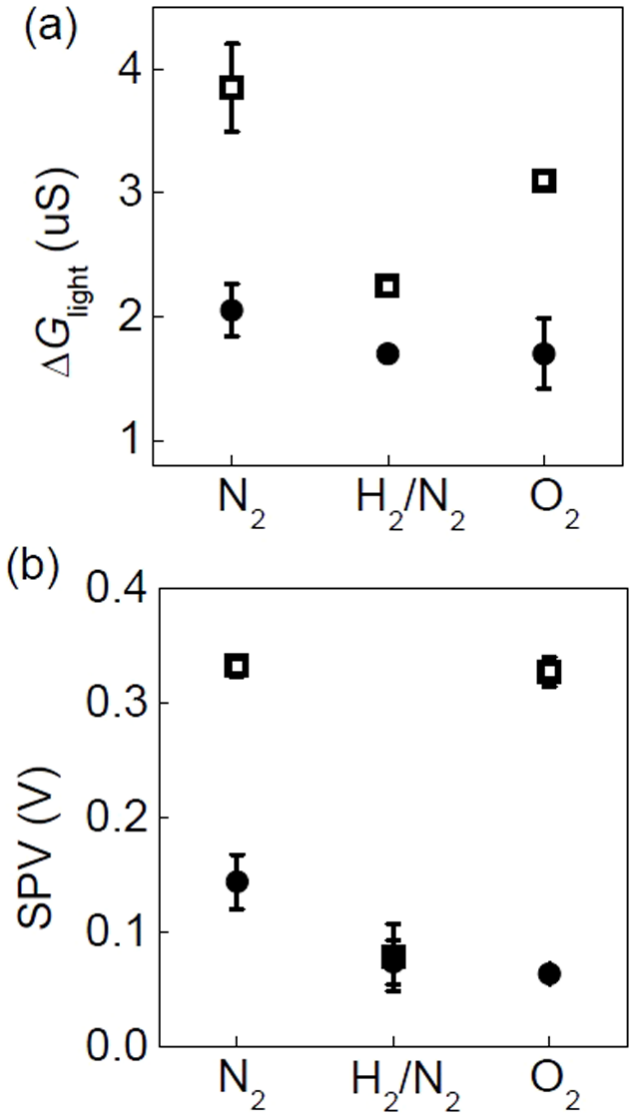
UV response of LAO/STO (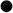) and Pd/LAO/STO (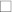) in N_2_, H_2_/N_2_, and O_2_: (a) Conductance difference, *ΔG*_light_ = *G*_light _− *G*_dark_, and (b) SPV = *W*_dark _− *W*_light_.

**Figure 7:**
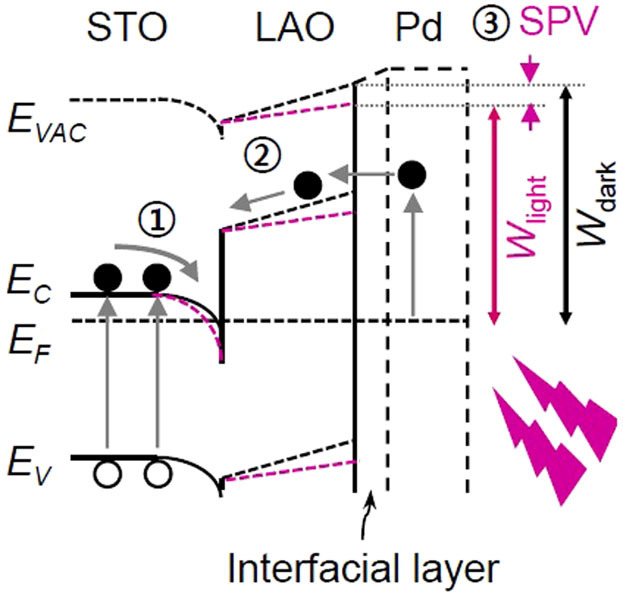
A schematic band diagram for Pd/LAO/STO under illumination of photon energy higher than bandgap of STO. 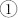
 Electron-hole pair generation in STO and 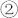 carrier excitation from Pd NPs. 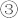 Resulting light-induced work function change, SPV = *W*_dark_ − *W*_light_.
